# Supplementary material for: Mycorrhizal types influence island biogeography of plants
Source: Commun Biol. 2021 Sep 24;4:1128. doi: 10.1038/s42003-021-02649-2 (PMC8463580; doi:10.1038/s42003-021-02649-2)
Supplement: Supplementary file 2 — Supplementary Information [file 42003_2021_2649_MOESM2_ESM.pdf]

Mycorrhizal Types Influence Island Biogeography of Plants

Supplementary Table 1. Multinomial model results.

Multinomial model explaining the probability of mycorrhizal types as a function of land type (mainlands and oceanic islands), absolute latitude, area, elevation range, and species richness. $*P < 0.05$, $**P < 0.01$, $***P < 0.001$.

Supplementary Table 1						
M1: Native Model (N = 1,273,566)						
	(Intercept)	land type (oceanic)	absolute latitude	area	elevation range	species richness
AM	0.901***	-0.088***	-0.23***	-0.003	-0.017***	0.028***
EM	-0.927***	0.221***	-0.16***	0.049***	-0.145***	0.099***
ORC	-1.872***	1.152***	-0.709***	-0.238***	0.124***	0.529***
M2: Naturalized Model (N = 121,886)						
AM	0.505***	0.07**	-0.196***	-0.06***	-0.016	0.046**
EM	-1.753***	0.295***	-0.137***	-0.157***	0.017	0.148***
ORC	-6.279***	2.093***	-1.081***	0.387**	-0.2*	0.339*

Supplementary Tables 2-6.

In our first set of models (M1 and M2 in each table), we compared the species richness patterns of plants with differing mycorrhizal associations. For each comparison, separate models were run for native and naturalized plants to predict plant species richness. In these generalized linear mixed effects models (GLMMs), we used a Poisson distribution because the response variable, species richness, is count data. The fixed effects were mycorrhizal status, land type (mainland, non-oceanic island or oceanic island) and their interaction; we also included the covariates of log-transformed absolute latitude, area, elevation, and plant species richness. The random effects were region, nested within land type and its interaction with mycorrhizal status. These random terms control for the non-independence of individual plant species records within floras, thereby providing general tests for differences in proportion of mycorrhizal species across island and mainland floras.

In our second set of models (M3, M4, M5, M6 in each table), we investigated geographical and environmental drivers of mycorrhizal status for native and naturalized plants in mainland and oceanic island floras. For each model, we used a composite response variable with species richness of each of the two mycorrhizal categories of interest to account for differences in species richness. For these analyses, we used generalized linear models (GLMs) with a logit link function, assuming a binomial distribution of the response variable. For these models, we took the natural logarithm of area, human population density, distance to the nearest mainland, elevation range and island age to normalize distributions. For the native mainland models, we included area, mean annual precipitation, mean annual temperature and elevation range. For the native island models, we included the same four variables with the addition of island age, island age squared and distance to the mainland.

Below we report the results from each of these sets of models (M1-M6) for analyses of the proportion of plant species in the floras that are ectomycorrhizal versus arbuscular mycorrhizal (EEM:AM) plants (Supplementary Table 2), orchid mycorrhizal versus AM or EEM (ORC:M) plants (Supplementary Table 3), AM versus non-mycorrhizal (AM:NM) plants (Table S4), EEM versus NM (EEM:NM) plants (Supplementary Table 5), and ORC versus NM (ORC:NM) plants (Supplementary Table 6). To ensure robust interpretation of patterns in the floras in the face of species with ambiguous mycorrhizal status, we repeated these models (M1-M6) for each combination of assigning ambiguous AMEEM plants to AM or EM, and the ambiguous AMNM plants as AM or NM. Four combinations of these ambiguous designations are arranged as follows: (a) AMEEM plants assigned to EEM and AMNM plants assigned to NM, (b) AMEEM plants assigned to EEM and AMNM plants assigned to AM, (c) AMEEM plants assigned to AM and AMNM plants assigned to NM, and (d) AMEEM plants assigned to AM and AMNM plants assigned to AM. In all cases, the results reported in the main text correspond to models that assign AMEEM plants to EEM and AMNM plants to NM.

For models M1 and M2 in each table, the table lists the reported estimate relative to the reference category along with a p value range of significance (*$P < 0.05$, **$P < 0.01$, ***$P < 0.001$). Note that these estimates are on the logit scale, reflecting the appropriate transformation for statistical analysis (see figures for back-transformed representations of the results). The reference for M1 and M2 for land type is always mainland, the reference for M1 and M2 for mycorrhizal status depends on the model, but is the mycorrhizal status not listed in parentheses. We are particularly interested in whether the proportion of mycorrhizal plant species in the island floras differ from that in the mainland floras. This effect is tested in the 'mycorrhizal status*land

type' interaction term. In Supplementary Table 1 M1 A, the estimate for the 'mycorrhizal status*land type (oceanic)' is 0.147 and is significant at p = 1.73e-10. This represents how much greater the proportion of EEM compared to AM plant species are in oceanic island floras relative to their proportion in mainland floras.

Supplementary Table 2. GLM explaining relative proportion EEM and AM (EEM:AM) plant species.

(1A) AMEEM plants assigned to EEM and AMNM plants assigned to NM, (1B) AMEEM plants assigned to EEM and AMNM plants assigned to AM, (1C) AMEEM plants assigned to AM and AMNM plants assigned to NM, and (1D) AMEEM plants assigned to AM and AMNM plants assigned to AM. The models in (1A) are those reported in the manuscript. $*P < 0.05$, $**P < 0.01$, $***P < 0.001$.

Supplementary Table 2				
M1: EEM:AM Native Model (GLMM), N = 1925				
	A	B	C	D
(intercept)	5.436***	5.796***	5.546***	5.83***
mycorrhizal status (EEM)	-1.506***	-1.779***	-1.649***	-1.915***
land type (non-oceanic)	1.199***	1.051***	1.027***	1.013***
land type (oceanic)	0.619***	0.512***	0.471***	0.478***
absolute latitude	-0.226***	1.371***	1.414***	1.358***
area	0.346***	0.000	-0.001	0.001
elevation range	0.161***	0.35***	0.309***	0.343***
species richness	1.448***	0.138***	0.145***	0.145***
mycorrhizal status*land type (non-oceanic)	0.013	-0.021	0.075**	0.038
mycorrhizal status*land type (oceanic)	0.147***	0.086***	0.177***	0.115***
M2: EEM:AM Naturalized Model (GLMM), N = 1148				
(intercept)	3.805***	4.186***	3.817***	4.192***
mycorrhizal status (EEM)	-1.9***	-2.28***	-2.012***	-2.382***
land type (non-oceanic)	1.048***	1.015***	1.076***	1.043***
land type (oceanic)	1.474***	1.382***	1.506***	1.414***
absolute latitude	1.052***	1.01***	1.053***	1.009***
area	-0.001	-0.001	-0.002	-0.001
elevation range	0.173	0.218.	0.183	0.232.
species richness	0.092	0.093	0.087	0.088

mycorrhizal status*land type (non-oceanic)	0.214**	0.31***	0.115.	0.206**
mycorrhizal status*land type (oceanic)	0.282***	0.356***	0.17***	0.233***
M3: EEM:AM Mainland Native Model (GLM), N = 515				
(intercept)	-1.426***	-1.715***	-1.583***	-1.864***
area	0.049***	0.049***	0.036***	0.037***
absolute latitude	0.115***	0.123***	0.049***	0.059***
squared latitude	0.027.	-0.042**	0.096***	0.027.
precipitation	0.004	0.008*	-0.005	-0.001
temperature	0.05***	0.067***	0.058***	0.075***
elevation range	-0.073***	-0.075***	-0.054***	-0.057***
spatial autocovariate	0.062***	0.06***	0.067***	0.067***
M4: EEM:AM Oceanic Island Native Model (GLM), N = 313				
(intercept)	-1.427***	-1.737***	-1.516***	-1.821***
area	0.171***	0.188***	0.097***	0.118***
distance	0.000	-0.012	0.002	-0.009
precipitation	0.036**	0.047***	0.019	0.03*
absolute latitude	0.135***	0.154***	0.052	0.075.
squared latitude	-0.146**	-0.226***	-0.118*	-0.198***
temperature	0.019	0.061.	-0.071*	-0.028
elevation range	-0.084***	-0.079***	-0.092***	-0.089***
spatial autocovariate	0.098***	0.097***	0.124***	0.12***
M5: EEM:AM Mainland Naturalized Model (GLM), N = 287				
(intercept)	-1.825***	-2.195***	-1.977***	-2.341***
area	-0.122***	-0.133***	-0.075**	-0.088***
population density	0.000*	0.000**	0.000	0.000
absolute latitude	0.229*	0.252**	0.186.	0.21*
squared latitude	-0.107	-0.139.	-0.049	-0.083
precipitation	0.025	0.045*	0.027	0.046*
temperature	-0.016	0.041	0.001	0.056
elevation range	0.053**	0.062***	0.044*	0.053**
spatial autocovariate	0.153***	0.151***	0.169***	0.167***
M6: EEM:AM Oceanic Island Naturalized Model (GLM), N = 100				
(intercept)	-1.668***	-1.96***	-1.836***	-2.121***
area	0.193***	0.193***	0.095.	0.1.
distance	-0.052	-0.041	-0.158***	-0.15***
precipitation	0.037	0.049	0.078*	0.091**
population density	0.000	0.000	0.000	0.000

absolute latitude	0.115	0.056	-0.049	-0.105
squared latitude	-0.453*	-0.476*	-0.161	-0.188
temperature	0.051	0.071	0.053	0.073
elevation range	-0.141**	-0.153***	-0.119*	-0.13*
spatial autocovariate	0.115*	0.11*	0.189**	0.185**

Supplementary Table 3. GLM explaining proportion Orchid versus EEM and AM mycorrhizal (ORC:M) plant species.

(2A) AMNM plants assigned to NM and (2B) AMNM plants assigned to M. The models in (2A) are those reported in the manuscript. $*P < 0.05$, $**P < 0.01$, $***P < 0.001$.

Supplementary Table 3		
M1: ORC:M Native Model (GLMM), N = 2212		
	A	B
(intercept)	5.356***	5.625***
mycorrhizal status (ORC)	-3.634***	-3.863***
land type (non-oceanic)	1.614***	1.586***
land type (oceanic)	1.07***	1.068***
absolute latitude	-0.37***	1.668***
area	1.719***	-0.319***
elevation range	0.255***	0.269***
species richness	0.348***	0.353***
mycorrhizal status*land type (non-oceanic)	-0.087	-0.122
mycorrhizal status*land type (oceanic)	-0.143.	-0.202**
M2: ORC:M Naturalized Model (GLMM), N = 2212		
(intercept)	-0.391	-0.335
mycorrhizal status (ORC)	-6.905***	-7.291***
land type (non-oceanic)	0.271	0.208
land type (oceanic)	0.415	0.287
absolute latitude	-0.826*	-0.961*
area	-1.135***	-1.167***
elevation range	3.927***	4.185***
species richness	-0.822***	-0.872***
mycorrhizal status*land type (non-oceanic)	1.372**	1.484***
mycorrhizal status*land type (oceanic)	0.954***	1.038***
M3: ORC:M Mainland Native Model (GLM), N = 486		
(intercept)	-3.211***	-3.446***
area	-0.068***	-0.483***
absolute latitude	-0.504***	-0.183***
squared latitude	-0.115***	0.147***
precipitation	0.147***	-0.301***
temperature	-0.32***	0.203***
elevation range	0.215***	0.077***
spatial autocovariate	0.077***	0.077***
M4: ORC:M Oceanic Island Native Model (GLM), N = 177		

(intercept)	-3.169***	-3.414***
area	0.293***	0.325***
distance	0.098***	0.093***
absolute latitude	-0.156*	-0.13*
squared latitude	-0.08	-0.135
precipitation	0.404***	0.408***
temperature	-0.146*	-0.101
elevation range	0.039	0.029
spatial autocovariate	0.154***	0.157***
M5: ORC:M Mainland Naturalized Model (GLM), N = 71		
(intercept)	-5.341***	-5.655***
area	-0.058	-0.071
population density	-0.001	-0.001
absolute latitude	-1.974***	-1.966***
squared latitude	1.584	1.574
precipitation	0.101	0.103
temperature	0.229	0.284
elevation range	-0.138	-0.134
spatial autocovariate	1.013***	1.02***
M6: ORC:M Oceanic Island Naturalized Model (GLM), N = 27		
(intercept)	-4.701***	-4.924***
area	-0.442	-0.485
distance	0.302	0.32
absolute latitude	0.096	0.125
squared latitude	-0.09	-0.245
precipitation	0.013	0.022
population density	0.001	0.002.
temperature	0.137	0.063
elevation range	0.057	0.051
spatial autocovariate	0.839.	0.875.

Supplementary Table 4. GLM explaining proportion of AM versus Non-mycorrhizal (AM:NM) plant species.

(3A) AMEEM plants assigned to AM and AMNM plants assigned to NM, (3B) AMEEM plants assigned to AM and AMNM plants assigned to AM, (3C) AMEEM plants assigned to EEM and AMNM plants assigned to NM, and (3D) AMEEM plants assigned to EEM and AMNM plants assigned to AM. The models in (3A) are those reported in the manuscript. *$P < 0.05$, **$P < 0.01$, ***$P < 0.001$.

Supplementary Table 4				
M1: AM:NM Native Model (GLMM), N = 1941 (A,C,D); N = 1938 (B)				
	A	B	C	D
(intercept)	5.532***	5.53***	5.51***	5.51***
mycorrhizal status (AM)	-0.756***	-1.757***	-0.728***	-0.728***
land type (non-oceanic)	1.016***	1.034***	1.018***	1.018***
land type (oceanic)	0.44***	0.458***	0.434***	0.434***
absolute latitude	-0.117***	1.359***	1.345***	1.345***
area	1.35***	-0.149***	-0.119***	-0.119***
elevation range	0.327***	0.323***	0.323***	0.323***
species richness	0.213***	0.208***	0.216***	0.216***
mycorrhizal status*land type (non-oceanic)	0.155***	0.143***	0.143***	0.143***
mycorrhizal status*land type (oceanic)	0.229***	0.212***	0.224***	0.224***
M2: AM:NM Naturalized Model (GLMM), N = 1181 (A,C,D); N = 1160 (B)				
(intercept)	3.79***	3.896***	3.893***	3.893***
mycorrhizal status (AM)	-0.356***	-1.348***	-0.342***	-0.342***
land type (non-oceanic)	1.171***	0.967***	0.968***	0.968***
land type (oceanic)	1.5***	1.333***	1.277***	1.277***
absolute latitude	0.976***	0.949***	0.912***	0.912***
area	-0.001	0.228***	0.277***	0.277***
elevation range	0.297*	0.203	0.22.	0.22.
species richness	0.1	0.083	0.093	0.093
mycorrhizal status*land type (non-oceanic)	-0.298***	-0.243***	-0.284***	-0.284***
mycorrhizal status*land type (oceanic)	-0.232***	-0.16***	-0.214***	-0.214***

M3: AM:NM Mainland Native Model (GLM), N = 515				
(intercept)	0.705***	1.71***	0.674***	0.674***
area	0.004.	0.000	0.001	0.001
absolute latitude	-0.018.	-0.013	-0.029**	-0.029**
squared latitude	-0.189***	-0.171***	-0.179***	-0.179***
precipitation	0.037***	0.071***	0.037***	0.037***
temperature	0.06***	0.026***	0.061***	0.061***
elevation range	-0.021***	-0.019***	-0.016***	-0.016***
spatial autocovariate	0.051***	0.073***	0.051***	0.051***
M4: AM:NM Oceanic Island Native Model (GLM), N = 325 (A,C, D); N = 323 (B)				
(intercept)	0.576***	1.587***	0.56***	0.56***
area	0.08***	0.06***	0.062***	0.062***
distance	-0.034**	-0.006	-0.032**	-0.032**
absolute latitude	0.036	0.067	0.02	0.02
squared latitude	-0.209***	-0.263***	-0.206***	-0.206***
precipitation	0.057***	0.074***	0.052***	0.052***
temperature	0.194***	0.125***	0.172***	0.172***
elevation range	0.032*	0.056*	0.032*	0.032*
spatial autocovariate	0.105***	0.148***	0.11***	0.11***
M5: AM:NM Mainland Naturalized Model (GLM), N = 294				
(intercept)	0.383***	1.379***	0.362***	0.362***
area	-0.051***	-0.062***	-0.041***	-0.041***
population density	0.000***	0.000**	0.000**	0.000**
absolute latitude	0.074	0.105	0.064	0.064
squared latitude	-0.102*	-0.133*	-0.092.	-0.092.
precipitation	0.068***	0.088***	0.067***	0.067***
temperature	0.185***	0.18***	0.187***	0.187***
elevation range	0.026*	0.027.	0.025*	0.025*
spatial autocovariate	0.125***	0.17***	0.127***	0.127***
M6: AM:NM Oceanic Island Naturalized Model (GLM), N = 109 (A,C,D); N = 105 (B)				
(intercept)	0.649***	1.591***	0.627***	0.627***
area	0.035	0.041	0.016	0.016
distance	0.034	0.005	0.022	0.022
absolute latitude	-0.36***	-0.255*	-0.379***	-0.379***
squared latitude	0.156	0.101	0.196	0.196
precipitation	0.055*	0.046	0.059*	0.059*
population density	0.000	0.000	0.000	0.000
temperature	0.138	0.171	0.13	0.13
elevation range	-0.052	-0.042	-0.047	-0.047

spatial autocovariate	0.091**	0.092	0.096**	0.096**

Supplementary Table 5. GLM explaining proportion of EEM versus non-mycorrhizal (EEM:NM) plant species.

(4A) AMEEM plants assigned to EEM and AMNM plants assigned to NM, (4B) AMEEM plants assigned to EEM and AMNM plants assigned to AM, (4C) AMEEM plants assigned to AM and AMNM plants assigned to NM, and (4D) AMEEM plants assigned to AM and AMNM plants assigned to AM. The models in (4A) are those reported in the manuscript. **P* < 0.05, ***P* < 0.01, ****P* < 0.001.

Supplementary Table 5				
M1: EEM:NM Native Model (GLMM), N = 1924 (A,C); N = 1921(B,D)				
	A	B	C	D
(intercept)	4.022***	4.033***	3.924***	3.937***
mycorrhizal status (EEM)	0.779***	-0.223***	0.894***	-0.108***
land type (non-oceanic)	1.057***	1.059***	1.077***	1.077***
land type (oceanic)	0.614***	0.617***	0.611***	0.613***
absolute latitude	-0.061***	1.312***	1.307***	1.298***
area	1.32***	-0.079***	-0.061***	-0.082***
elevation range	0.384***	0.386***	0.372***	0.374***
species richness	0.16***	0.15***	0.167***	0.156***
mycorrhizal status*land type (non-oceanic)	0.124**	0.112**	0.075.	0.062.
mycorrhizal status*land type (oceanic)	0.074*	0.053.	0.047.	0.028
M2: EEM:NM Naturalized Model (GLMM), N = 1141 (A,C); N = 1120 (B,D)				
(intercept)	2.074***	2.107***	1.995***	2.037***
mycorrhizal status (EEM)	1.584***	0.583***	1.674***	0.674***
land type (non-oceanic)	1.143***	1.057***	1.047***	0.952***
land type (oceanic)	1.535***	1.536***	1.43***	1.423***
absolute latitude	0.803***	0.801***	0.787***	0.78***
area	0.375***	0.356***	0.388***	0.368***
elevation range	0.145	0.099	0.158	0.111
species richness	0.164*	0.158.	0.154.	0.143.
mycorrhizal status*land type (non-oceanic)	-0.585***	-0.504***	-0.496***	-0.411***

mycorrhizal status*land type (oceanic)	-0.568***	-0.481***	-0.462***	-0.375***
M3: EEM:NM Mainland Native Model (GLM), N = 515				
(intercept)	-0.753***	0.254***	-0.881***	0.125***
area	0.05***	0.049***	0.04***	0.038***
absolute latitude	0.083***	0.094***	0.033*	0.049**
squared latitude	-0.156***	-0.142***	-0.099***	-0.093***
precipitation	0.044***	0.078***	0.034***	0.07***
temperature	0.1***	0.065***	0.109***	0.073***
elevation range	-0.09***	-0.094***	-0.075***	-0.079***
spatial autocovariate	0.07***	0.082***	0.075***	0.082***
M4: EEM:NM Oceanic Island Native Model (GLM), N = 244				
(intercept)	-0.878***	0.15***	-0.935***	0.088***
area	0.241***	0.207***	0.178***	0.147***
distance	-0.04*	-0.004	-0.033.	0.002
absolute latitude	0.203***	0.177**	0.111.	0.084
squared latitude	-0.317***	-0.269*	-0.228*	-0.178
precipitation	0.096***	0.111***	0.075***	0.09***
temperature	0.307***	0.26***	0.245***	0.203***
elevation range	-0.038	-0.008	-0.041	-0.009
spatial autocovariate	0.138***	0.187***	0.155***	0.201***
M5: EEM:NM Mainland Naturalized Model (GLM), N = 287				
(intercept)	-1.462***	-0.463***	-1.595***	-0.596***
area	-0.172***	-0.194***	-0.129***	-0.149***
population density	0.000***	0.000***	0.000**	0.000***
absolute latitude	0.28**	0.319**	0.241*	0.278*
squared latitude	-0.184*	-0.227*	-0.132	-0.175.
precipitation	0.094***	0.111***	0.092***	0.109***
temperature	0.175***	0.168***	0.189***	0.18***
elevation range	0.081***	0.083***	0.074***	0.076**
spatial autocovariate	0.167***	0.195***	0.183***	0.21***
M6: EEM:NM Oceanic Island Naturalized Model (GLM), N = 90 (A,C); N = 89 (B,D)				
(intercept)	-1.111***	-0.164**	-1.261***	-0.313***
area	0.161**	0.173*	0.108.	0.122
distance	-0.035	-0.072	-0.133*	-0.167**
absolute latitude	0.37*	0.424.	0.274	0.317
squared latitude	-1.222***	55.**	-1.068**	-1.038**
precipitation	0.064.	0.064	0.102*	0.1*
population density	0.000	0.000***	0.000***	0.000***
temperature	0.242	0.262	0.215	0.243
elevation range	-0.162**	-0.157*	-0.161*	-0.156*
spatial autocovariate	0.078.	0.087	0.116.	0.123

Supplementary Table 6. GLM explaining the proportion orchid versus non-mycorrhizal (ORC:NM) plant species.
$*P < 0.05$, $**P < 0.01$, $***P < 0.001$.

Supplementary Table 6	
M1: ORC:NM Native Model (GLMM), N = 2212	
(intercept)	3.517***
mycorrhizal status (ORC)	-1.704***
land type (non-oceanic)	1.593***
land type (oceanic)	1.11***
absolute latitude	-0.216***
area	1.588***
elevation range	0.28***
species richness	0.381***
mycorrhizal status*land type (non-oceanic)	-0.241*
mycorrhizal status*land type (oceanic)	-0.359***
M2: ORC:NM Naturalized Model (GLMM), N = 2212	
(intercept)	-0.581*
mycorrhizal status (ORC)	-5.468***
land type (non-oceanic)	0.426
land type (oceanic)	0.548
absolute latitude	-0.24
area	-0.634***
elevation range	2.774***
species richness	-0.565**
mycorrhizal status*land type (non-oceanic)	1.88***
mycorrhizal status*land type (oceanic)	1.406***
M3: ORC:NM Mainland Native Model (GLM), N = 486	
(intercept)	-1.283***
area	-0.037***
absolute latitude	-0.446***
squared latitude	-0.327***
precipitation	0.253***
temperature	-0.279***
elevation range	0.161***
spatial autocovariate	0.105***
M4: ORC:NM Oceanic Island Native Model (GLM), N = 177	
(intercept)	-1.26***
area	0.401***
distance	0.088**
absolute latitude	-0.109
squared latitude	-0.279**
precipitation	0.53***

temperature	-0.067
elevation range	-0.027
spatial autocovariate	0.213***
M5: ORC:NM Mainland Naturalized Model (GLM), N = 71	
(intercept)	-3.782***
area	-0.099
population density	-0.001
absolute latitude	-2.02***
squared latitude	1.604
precipitation	0.169
temperature	0.352
elevation range	-0.168
spatial autocovariate	1.106***
M6: ORC:NM Oceanic Island Naturalized Model (GLM), N = 27	
(intercept)	-2.854***
area	-0.596
distance	0.353
absolute latitude	0.37
squared latitude	-0.863
precipitation	0.009
population density	0.002.
temperature	-0.147
elevation range	-0.011
spatial autocovariate	1.002*

Supplementary Table 7. GLM explaining the proportion ectomycorrhizal versus arbuscu lar (EEM:AM) and versus non-mycorrhizal (EEM:NM) plant species with latitude.

GLM explaining the proportion ectomycorrhizal versus arbuscular (EEM:AM) and versus non-mycorrhizal (EEM:NM) plant species as a function of latitude and squared latitude in mainlands and oceanic islands.
$*P < 0.05$, $**P < 0.01$, $***P < 0.001$.

Supplementary Table 7	
M1: EEM:AM Mainland Native Model (GLMM), N = 515	
(intercept)	-1.442***
absolute latitude	0.138***
squared latitude	-0.043***
spatial autocovariate	0.057***
M2: EEM:AM Oceanic Island Native Model (GLMM), N = 264	
(intercept)	-1.334***
absolute latitude	0.129**
squared latitude	-0.165***
spatial autocovariate	0.103***
M3: EEM:NM Mainland Native Model (GLMM), N = 515	
(intercept)	-0.776***
absolute latitude	0.031**
squared latitude	-0.213***
spatial autocovariate	0.066***
M4: EEM:NM Native Model (GLMM), N = 264	
(intercept)	-0.621***
absolute latitude	0.245***
squared latitude	-0.473***
spatial autocovariate	0.131***

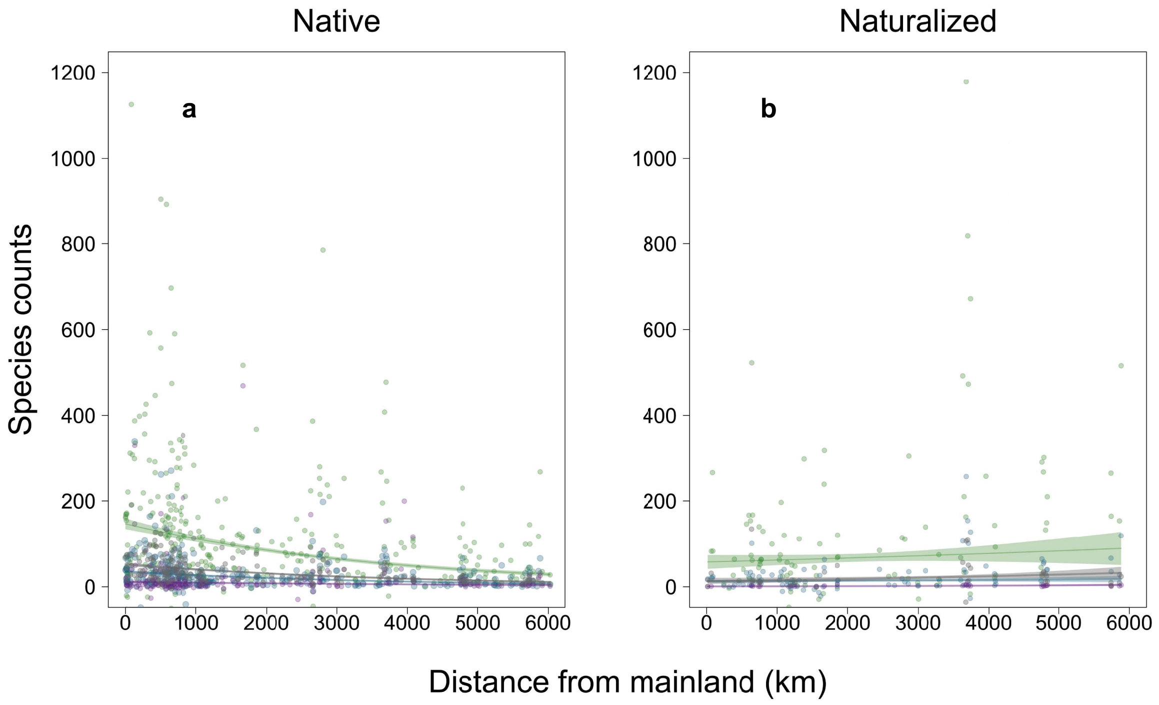

Supplementary Fig. 1. Plant species counts in native and naturalized oceanic island flora with distance.

The proportion of AM:NM plants in the native island flora decreases with oceanic island distance from the mainland (**a** estimate = -0.034 ± -0.006, $p < 0.01$, n = 325; GLM), consistent with AM plants being differentially limited in colonization of far islands. In contrast, no patterns with distance are detectable in naturalized oceanic island floras (**b** estimate = 0.034 ± 0.005, $p = 0.25$, n = 105; GLM).

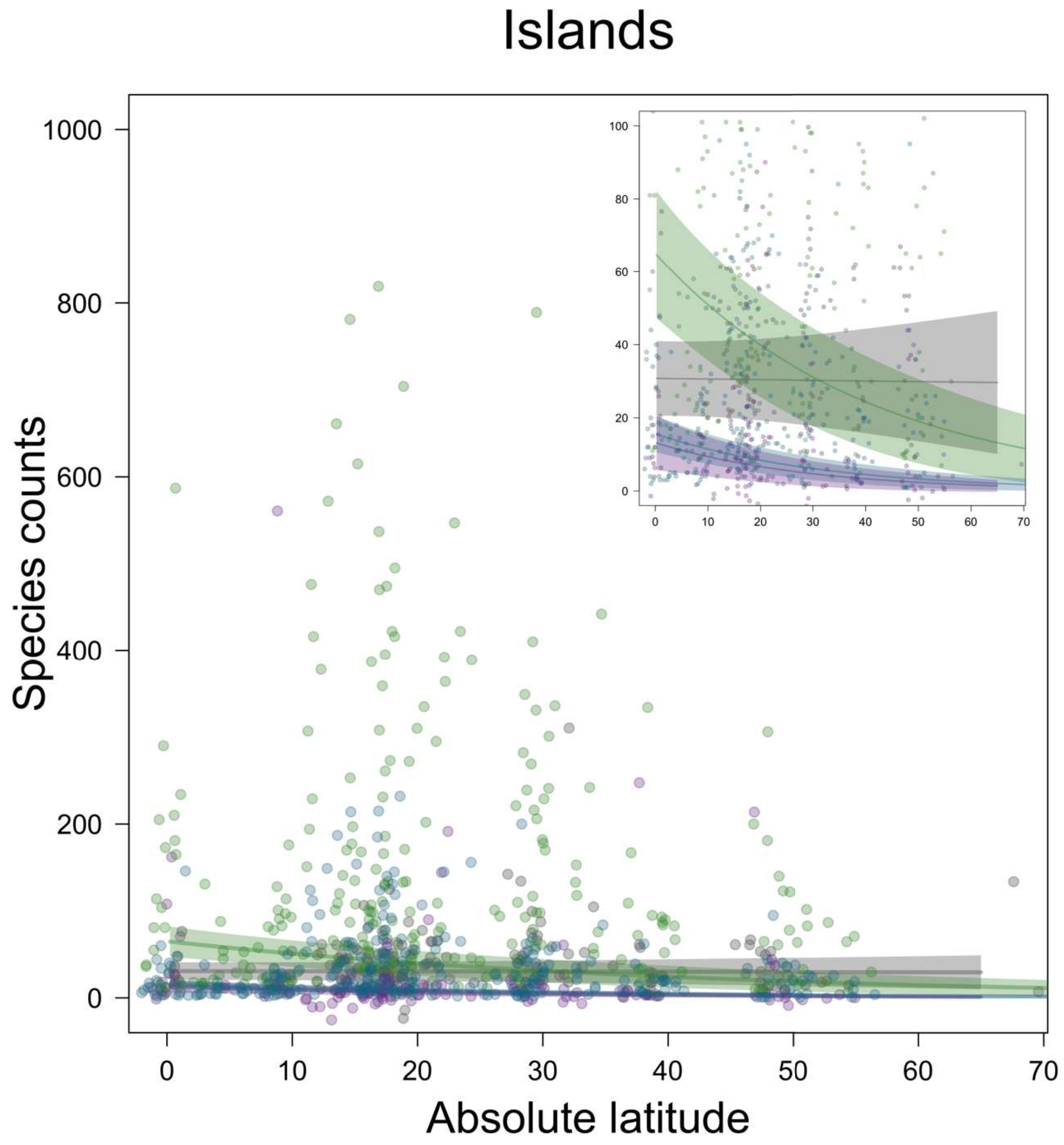

Supplementary Fig. 2. Latitudinal pattern in native oceanic island floras.

The latitudinal plant species gradient is not as strongly influenced by type of mycorrhizal plant species as in mainland plant species. In island regions, the proportion of mycorrhizal plant species decreases with absolute latitude (º from equator) more strongly for arbuscular mycorrhizal (AM; green line: absolute latitude estimate = 0.067 ± 0.012, $p < 0.001$, n = 264; squared latitude estimate -0.001 ± 0.000, $p < 0.001$, n = 264; GLM), than for ectomycorrhizal plant species (EEM; blue line: absolute latitude estimate = 0.080 ± 0.014, $p < 0.01$, n = 264; squared latitude estimate = -0.002 ± 0.000, $p < 0.001$, n = 264; GLM) and orchid mycorrhizal plant species (ORC; purple line: absolute latitude estimate = 0.025 ± 0.024, $p = 0.28$, n = 158; squared latitude estimate = -0.001 ± 0.000, $p = 0.03$, n = 158). Non-mycorrhizal species counts plotted for refer-

ence (NM; grey line: absolute latitude estimate = 0.014 ± 0.014, $p = 0.30$, n = 158; squared latitude estimate = -0.000 ± 0.000, $p = 0.34$, n = 158; GLM). The Insert shows the relationship for a limited span of the y axis (0 to 100 species) for clarity.

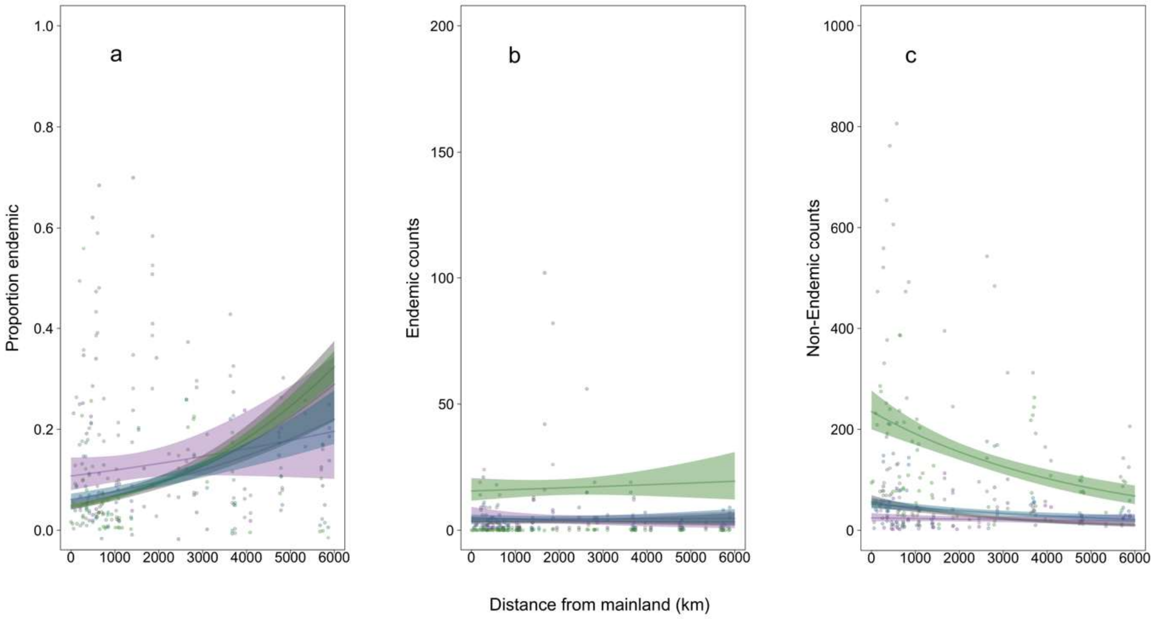

Supplementary Fig. 3 Endemic variation in mycorrhizal types in oceanic island floras with distance from mainland source regions.
The proportion of oceanic island plant species endemic to non-endemic increases most rapidly with distance for AM plant species (**a** estimate = 0.432 ± 0.063, $p < 0.001$, n = 254; GLM). The number of endemic AM species does not change with distance (**b** estimate 0.048 ± 0.147, p = 0.74, n = 254; GLM). The non-endemic species for AM decreases most strongly compared to other mycorrhizal types and to NM plants (**c** estimate = -0.265 ± 0.067, $p < 0.001$, n = 254; GLM).

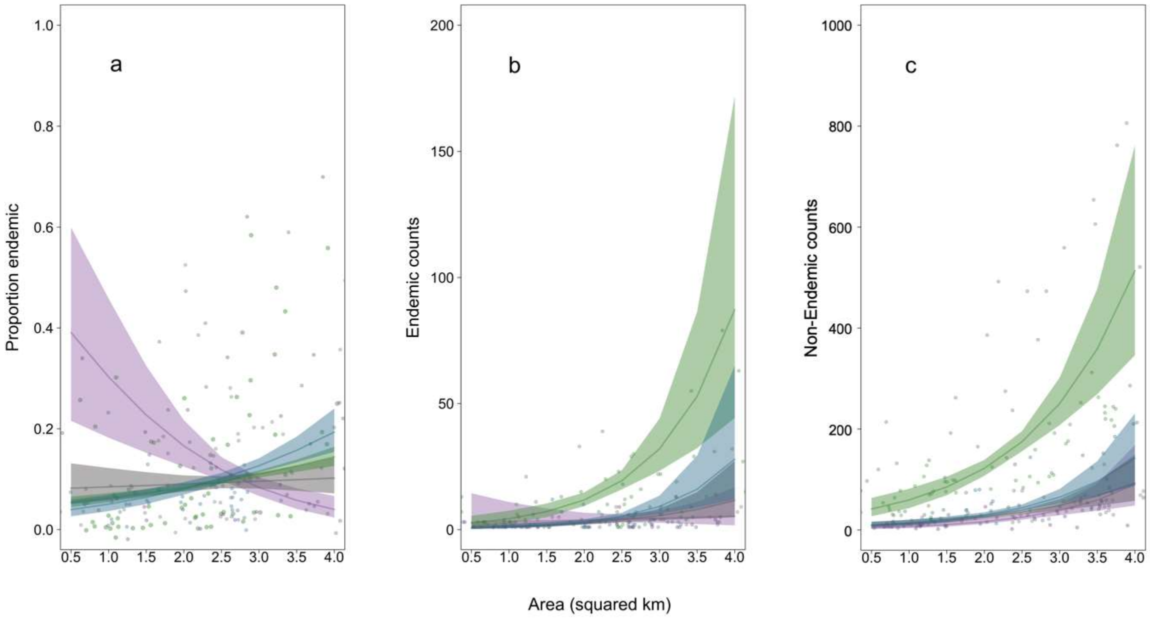

Supplementary Fig. 4. Endemic variation in mycorrhizal types in oceanic island floras with area.

The proportion of oceanic island plant species endemic to non-endemic increases least with island area for NM plant species (**a** estimate = -0.338 ± 0.122, $p < 0.01$, n = 254; GLM) and decreases for ORC plant species (estimate = -0.998 ± 0.165, $p < 0.001$, n=254, GLM). Both the number of endemic species and non-endemic species increase with area (**b, c** endemic: estimate 0.866 ± 0.200, $p < 0.001$, n = 254, GLM; non-endemic: estimate 0.608 ± 0.113, $p < 0.001$, n = 254, GLM) for all mycorrhizal types except ORC endemic species. For endemic species, ORC plant species richness decreases with area (estimate -0.734 ± 0.355, p = 0.04, n = 254, GLM).

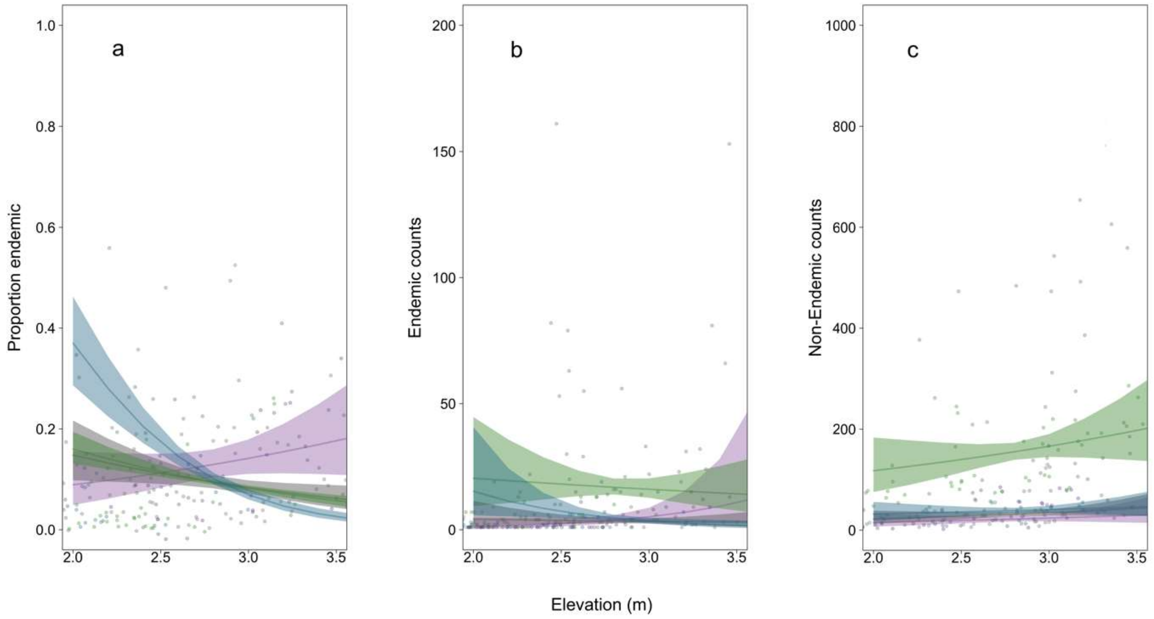

Supplementary Fig. 5. Endemic variation in mycorrhizal types in oceanic island floras with elevation.

The proportion of oceanic island of plant species endemic to non-endemic decreases most strongly with elevation for EEM plant species (**a** estimate = -0.705 ± 0.080, $p < 0.001$, n = 254; GLM). The number of endemic species decreases with elevation (**b** estimate -0.503± 0.200, p = 0.01, n = 254) for all mycorrhizal types except ORC plants, which increase in species richness with elevation (estimate 1.007 ± 0.346, $p < 0.01$, n = 254, GLM). The number of non-endemic species shows no relationship with elevation across mycorrhizal types (estimate 0.082 ± 0.113, p = 0.47, n = 254, GLM).
