# Supplementary material for: Mycorrhizal types influence island biogeography of plants
Source: Commun Biol. 2021 Sep 24;4:1128. doi: 10.1038/s42003-021-02649-2 (PMC8463580; doi:10.1038/s42003-021-02649-2)
Supplement: Supplementary file 3 — Description of Additional Supplementary Files [file 42003_2021_2649_MOESM3_ESM.pdf]

Description of Additional Supplementary Files

File name: Supplementary Data 1.
Description: Table of families and corresponding consensus proportions of mycorrhizal status. Consensus proportions were calculated by averaging each mycorrhizal status proportion within each family across the three status references.
